# Identification and Characterization of Renal Cell Carcinoma Gene Markers

**Published:** 2007-02-09

**Authors:** Gul S. Dalgin, Dustin T. Holloway, Louis S. Liou, Charles DeLisi

**Affiliations:** 1 Molecular Biology, Cell Biology and Biochemistry Program, Boston University, 2 Cummington Street, Boston, MA 02215, U.S.A; 2 Department of Urology, Boston University School of Medicine, 715 Albany Street, Boston, MA 02118, U.S.A; 3 Biomedical Engineering, Boston University, 24 Cummington Street, Boston, MA 02215, U.S.A; 4 Bioinformatics and Systems Biology, Boston University, 24 Cummington Street, Boston, MA 02215, U.S.A

**Keywords:** Cancer diagnosis, biomarker identification, microarray analysis, Renal cell carcinoma

## Abstract

Microarray gene expression profiling has been used to distinguish histological subtypes of renal cell carcinoma (RCC), and consequently to identify specific tumor markers. The analytical procedures currently in use find sets of genes whose average differential expression across the two categories differ significantly. In general each of the markers thus identified does not distinguish tumor from normal with 100% accuracy, although the group as a whole might be able to do so. For the purpose of developing a widely used economically viable diagnostic signature, however, large groups of genes are not likely to be useful. Here we use two different methods, one a support vector machine variant, and the other an exhaustive search, to reanalyze data previously generated in our Lab (Lenburg et al. 2003). We identify 158 genes, each having an expression level that is higher (lower) in every tumor sample than in any normal sample, and each having a minimum differential expression across the two categories at a significance of 0.01. The set is highly enriched in cancer related genes (p = 1.6 × 10^−12^), containing 43 genes previously associated with either RCC or other types of cancer. Many of the biomarkers appear to be associated with the central alterations known to be required for cancer transformation. These include the oncogenes JAZF1, AXL, ABL2; tumor suppressors RASD1, PTPRO, TFAP2A, CDKN1C; and genes involved in proteolysis or cell-adhesion such as WASF2, and PAPPA.

## 1. Introduction

Renal-cell carcinoma (RCC) is the most common kidney neoplasm, comprising 3% of all adult malignancies (Jemal et al. 2003). Its incidence has increased steadily over the past 20 years in the United States and Europe; 35,000 new cases and 12,000 deaths now occur annually in the United States alone. Histopathologically, about 60–70% of RCC is clear-cell type (cc-RCC). Small and localized tumors are generally asymptomatic; pain, flank mass, or hematuria, being generally associated with locally advanced or metastatic tumors. Diagnosis is confirmed by imaging, including X-ray and computed-tomography. The 5-year survival rate of metastatic RCC is less than 10%. Moreover, RCC is one of the most therapy-resistant carcinomas, responding very poorly or not at all to radiotherapy, hormonal therapy, and chemotherapy. All these facts emphasize the importance of developing early diagnostic markers.

Microarray gene expression profiling has been used by ourselves (Lenburg et al. 2003) and others (Young et al. 2001; Boer et al. 2001; Gieseg et al. 2002; Young et al. 2003; Yamazaki et al. 2003; Lenburg et al. 2003; Higgins et al. 2003; Takahashi et al. 2003; Sultmann et al. 2005; Jones et al. 2005) to distinguish the various histological subtypes of RCC, and consequently to identify novel tumor markers. The general procedure identifies markers in accordance with average differential expression level (fold change) and/or some level of significance as measured by the t-test. Lenburg et al. used a 3-fold difference in expression and a level of significance of 0.03.

Here, we reanalyze the data of Lenburg et al. using a rigorous exhaustive search approach (Dalgin and DeLisi, 2005), and a more general, but approximate, approach based on support vector machines. We identify, by exhaustive search, 158 genes each of which (i) is consistently over- or under-expressed in all tumors and (ii) has a minimum expression level difference at better than 99% confidence. Sixty four of these *markers* were not identified previously (Lenburg et al. 2003). The set is highly enriched in cancer related genes (p = 1.6 × 10^−12^), containing 43 previously associated with either RCC or other types of cancer.

Among the set of genes that we identify as being related to RCC, some were known from previous studies (e.g. ATP6V1B1, EGLN3, SLC25A5, TUBB, ALDOA); others had never before been associated with RCC, but have been identified with other cancers (e.g. ABL2, JAZF1, TFAP2A). We identified biological roles of marker genes, and found pathways that are dominantly up-regulated (83% of immune response genes, all amino acid transport genes) or down-regulated (all cation and anion transport genes, all OXPHOS genes) which are related to kidney function (cation/anion transport genes) and RCC physiology (OXPHOS). Finally we constructed a model for RCC through functional classification of genes related to changes in cellular processes that are critical to initiation and progression of cancer.

## 2. Methods

### 2.0. Background

Briefly, (Lenburg et al. 2003) hybridized total RNA isolated from 9 clear-cell renal tumors and adjacent normal tissue (18 samples) to Affymetrix U133A and U133B arrays containing approximately 45,000 probe-sets. Of these, 27,609, representing 20,192 unigenes, gave a signal above background. Differentially expressed genes were identified by t-test and fold change. The average fold change was calculated as log_2_ (C/N) where C and N represent the average of tumor and normal expression values, respectively. Some 1706 probe-sets (1471 unique genes) were more than three-fold changed in renal tumors and had a p-value <0.03. Of these, 113 had been previously identified in three or more studies (Young et al. 2001; Boer et al. 2001; Takahashi et al. 2001; Gieseg et al. 2002). An obvious limitation of drawing conclusions from such a study is the small number of samples per category with a relatively large number of potential markers. We discuss this in detail below.

### 2.1. Identifying single gene biomarkers by exhaustive search

Here we identify single genes that correctly classify every sample, by direct comparison of differential expression in every tumor-normal pair (9^2^ comparisons per gene × 20,192 genes). A gene whose level of expression in every normal sample is always either greater or less than its level in every tumor sample, is ranked according to the smallest expression level distance across the two categories (Perl script is available at http://visant.bu.edu/skirca/script.pl). The smallest separation for a down (up)-regulated gene is the difference between the maximum (minimum) expression level in the tumor samples, and the minimum (maximum) expression level in the normal samples. In particular define *E_m,i_* as the expression level of the i^th^ gene in the m^th^ tumor sample and let *E_n,i_* be similarly defined for the normal samples. The minimum distance for the i^th^ gene is defined as

(1) $$d_{i}=\text{min}\left|\left\{E_{m,i}-E_{n,i}\right\}\right|$$

provided all differences have the same sign, where m and n range independently over all samples.

We identified 478 probes, corresponding to 466 unique genes, that separate all tumor from all normal tissue. Each gene was then tested for significance as follows.

**Figure 1.**
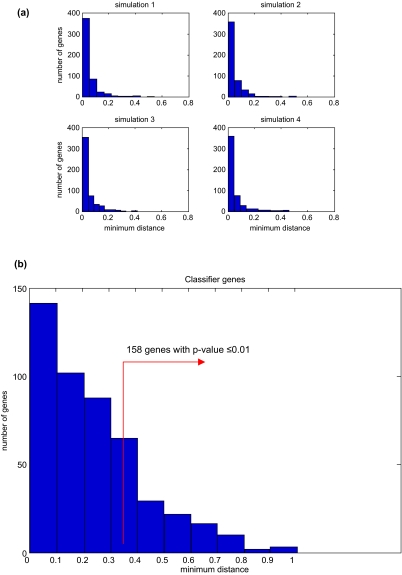
**(a)** The minimum distances of randomly formed expression profiles in four simulations are shown as representative of other simulations. The x-axis is the minimum distance and y-axis is the number of genes having that distance. **(b)** The distribution of minimum distances for 466 genes. 158 of these genes have minimum distances with p-values ≤0.01, hence identified as significant single gene biomarkers.

For a given gene we randomly selected 9 tumor expression values from all tumor expression values (20,192 genes × 9 tumor samples = 181,728 expression values), and 9 normal values from all normal expression values, subject to the constraint that all tumor values are greater (less) than all normal values. We calculated the minimum distance and repeated the procedure 500 times (to mimic random selection of 466 genes) to obtain a distribution of minimum distances (Figure 1(a)). The procedure was repeated 200 times to estimate the dispersion in the parameters of the sampling distribution. Overall, we obtained skew minimum distance distributions with an average mean minimum distance 0.0425 (standard deviation over 200 simulations = 0.00261) and average standard deviation 0.05768 (standard deviation over 200 simulations = 0.00531) across all simulations. The p-value for each actual minimum distance (d) was calculated using the total random data (500 × 200 = 10^5^) as probability of finding ≥d randomly (P(d_random_ ≥d). Of the initial set of 466 genes 158 were found to have significant minimum distances >0.28 with p-value ≤0.01 (Figure 1(b)). We refer to these as *significant single gene biomarkers* or simply *markers*.

### 2.2. SVM Recursive feature elimination

An alternative strategy for selecting markers is to use statistical feature selection techniques to rank genes. This can be done in a number of ways. Here we use a support vector machine (SVM) (Vapnik, 1998). which has been used effectively in other contexts (Holloway et al. 2005; Holloway et al. 2006a; Holloway et al. 2006b), and which has a well established statistical framework. The idea is again to find genes whose distance between tumor/normal sample expression values is in some sense maximum. Guyon et al. (Guyon et al. 2002) used a similar method to identify genes that stratify leukemia (Golub et al. 1999), and for distinguishing between colon cancer and normal tissue (Alon et al. 1999), by dividing the data sets into two equal halves for training and testing. They recursively discarded the features (genes) with smallest weight (see below for detailed discussion) to select genes that separate the two classes at a specified accuracy. For leukemia they discovered 2 genes that yield zero leave-one-out error. For colon cancer, they obtained 98% accuracy using 4 genes. The main difference here is the introduction of a procedure that does not assume the highest ranked gene is necessarily more useful than genes that rank slightly lower.

An SVM is an effective method for making predictions on many types of data including handwritten text, protein sequence, DNA sequence, and microarray profiles, when the number of data attributes for each sample is very large. Briefly, the method seeks to find a maximal separation between two training sets (Sholkopf and Smola, 2002), a positive set, in this case tumor samples; and a negative set, normal samples. Each sample is labeled by a set of attributes, here gene expression levels, and hence can be represented by a vector that can have tens of thousands of components. The separation between positive and negative vectors (samples) is achieved through an optimization which finds a hyperplane bisecting the two sets. The hyperplane must be as distant as possible from the two sets thus creating a *maximal-margin* separator (Holloway et al. 2005).

Most of the attributes are irrelevant to separation. The SVM algorithm can be used to rank the importance of the various attributes by a method referred to as recursive feature elimination (RFE), and thus identify those genes that are most discriminatory. An important SVM output is a vector of learned weights; each component of the vector being a weight of an attribute: the higher the weight the more useful in separating positives from negatives. The original SVM-RFE algorithm trains an SVM, calculates the weight of each attribute, and discards a specified number of low weight attributes (Guyon et al. 2002). The process is repeated until the desired number of attributes remains. Typically half the attributes are removed at each iteration until some threshold is reached after which only one at a time is removed.

The procedure employed here is different in that the entire SVM-RFE ranking is performed many times within a leave-one-out cross validation. Briefly, the procedure is as follows. Prior to applying the SVM, we use a t-test with a loose p-value threshold of 0.1 to filter the large majority of statistically irrelevant genes, leaving 10,479 genes for further analysis. We then perform SVM-RFE on *n*-1 samples, save the results, and repeat *n* times. Thus for the renal cancer dataset with n = 18 samples we ranked the performance of each gene 18 times (18 cross-validations). Intuitively, genes that are repeatedly ranked near the top are robust to changes in the training set and are considered more reliable. The fluctuation of gene choice during cross validation can be seen more clearly when examining a list showing the highest ranked gene on each training set of the leave-one-out procedure. On the 18 possible training sets, 12 different genes were given a rank of 1 at least once (data not shown). Because gene rank can change markedly with dataset, choosing consistent genes increases the chances that the chosen genes are truly reliable markers. *This highlights the need to choose genes that are consistent across samples, as opposed to genes whose average value differs significantly across the two categories.*

To derive our final gene ranking, we arbitrarily selected the top 20 genes from each ranking and counted the number of times each gene appears in this combined set. The maximum occurrence of 18 would indicate a very stable biomarker; i.e. one that was chosen by each training set. The final list represents genes that are stable across sample sets and can thus be considered reliable biomarkers.

## 3. Results

### 3.1. Single gene markers

#### Pathway/process enrichment

Of the 158 markers, 73 are annotated in a KEGG pathway and another 42 are in a GO process at level 5 or higher. The pathways/processes with the highest number of classifiers are shown in Table 1. The categories are biologically plausible, having already been implicated in cancer transformation (e.g. OXPHOS, apoptosis, cell adhesion, MAPK signaling) or being potentially important (calcium signaling pathway, fatty acid metabolism and cation transport) in transformation.

#### Marker Significance

**Figure 2.**
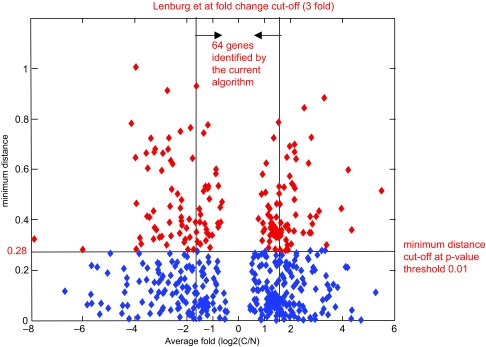
The relationship between minimum distance and average fold change (log2(C/N)). Average fold change was previously calculated by Lenburg et al. and the significance was found by t-test. Here, C and N denotes the average expression values in tumor and normal samples, respectively. Significant markers (p-value ≤0.01, 158 genes) are indicated as red. 64 genes are shown in between the vertical arrows. These genes have an average fold change less than 3 hence were not identified as previously differentially expressed. Yet, these genes have been identified as new potential biomarkers by the current algorithm.

We (Lenburg et al. 2003) previously identified 1471 genes that were more than three-fold changed in renal tumors with a p-value <0.03. The relationship between minimum distance determined by exhaustive search, and the average fold change, is shown in Figure 2. Of the 158 *markers* (indicated in red) 94 (59%) have changed at least 3 fold in tumor with a t-test p-value <0.03, and hence overlap with the 1471 genes identified by (Lenburg et al. 2003).

There are at least two reasons to expect that the entire set of markers, including the 64 not previously identified, are likely to be useful signatures. The first, as indicated above, is that they are not only uniformly over or under-expressed, but the magnitude of the minimum differential expression is statistically significant, taking into account of the small sample size (see Section 3.3. for further analysis). Second, the set (158) and subset (64) are both highly enriched in cancer associated genes. In particular, of the 158 markers, 43 (27.2%) have been previously reported to be cancer associated as indicated in the Genetic association database or OMIM. In addition, 15 of the 64 are found to be cancer associated (23.4%). In comparison with the OMIM database, which includes 1351 cancer related genes (8.1%) out of 16603 total genes, 158 classifier genes are enriched 3.3 times (Fisher’s exact test p-value = 1.6 × 10^−12^) with the cancer related genes; and 64 genes are enriched 2.9 times with p = 1. 6 × 10^−4^. Both results indicate that the enrichment of 158 genes and 64 genes with cancer genes is significant with respect to OMIM.

We also performed simulations by randomly selecting 158 genes from OMIM, and recorded the percentage of cancer associated genes. We tossed 1000 times, obtained a random distribution, and repeated the entire procedure 10 times to estimate the dispersion of the parameters of the 10 distributions. We obtained an average of 6.8% cancer related genes with a standard deviation 1.9 (The dispersion of both parameters is less than 2%). The actual percentage for 158 genes, 27.2%, is 10.7 standard deviations away from the random mean. We performed the same simulations by drawing randomly 64 genes, which yielded an average of 6.85% cancer related genes with a standard deviation 3.1. Hence, the actual percentage for 64 genes (23.2%) is 5.3 standard deviations away from the random mean. These results confirm that the percentage of cancer related marker genes are significantly different from what would be obtained randomly. Of the genes identified previously (Lenburg et al. 2003) 220 (16%) are cancer associated; i.e. the 64 new genes are enriched nearly 50% more than the original set.

### 3.2. Identifying biomarkers by SVM

The number of occurrences of the genes across the 18 top 20 rankings is given as Supplementary Figure 1. Each gene is shown by its rank (the highest ranked gene, i.e. gene #1, occurs in the top 20, 17 out of 18 times; the number 2 ranked genes has 16 occurrences, the genes ranked 3 and 4 have 14 occurrences etc). Examination of the genes most frequently ranked in the top 20 reveals that they can all individually distinguish tumor from normal tissue with no error. More importantly, these are the genes that are most stable over different choices of training set (18 training sets each lacks one sample), suggesting that they would be the most likely to make accurate predictions in unseen tissue samples.

Genes ranked in the top 20 (Table 2) were all identified as classifiers by the exhaustive search (Section 2.1), 17 with a p-value ≤0.01; i.e. 7/20 (85%) are in our set of 158 markers. Twelve of the genes were previously identified by Lenburg et al., two genes by four or more other RCC studies as well. HUGO (The Human Genome Organization) gene symbol (http://www.gene.ucl.ac.uk/nomenclature/) is used to represent the genes in Table 2 and throughout the paper.

Of the 20 genes, 7 had not been previously identified as RCC related, of those 5 are identified by both methods, and 2 of the five are implicated in other cancers.

### 3.3. Small sample size

Although we confined ourselves to perfect separators with minimum separation distances that are highly significant, human polymorphism makes it unlikely that perfect separation will continue to hold as the population size increases. In an effort to gain some insight into this effect we analyzed a breast cancer data set (Ma et al. 2003), which includes 32 normal samples and 53 breast cancer samples (30 ductal carcinoma *in situ* and 23 invasive ductal carcinoma samples). Raw data is available for 1940 genes which were found to be differentially expressed between normal and cancer stages by linear discriminant analysis (Ma et al. 2003).

We drew random groups of 18 (9 normal, 9 tumor) and repeated the analysis of 2.1. The significant markers were then tested on the remaining samples. The entire procedure was repeated 100 times. Denoting tumor samples as positives and normal samples as negatives, the following performance measures were used for the classifier genes: *sensitivity* = TP/(TP+FN); *specificity* = TN/(TN+FP) and *positive predictive value (PPV)* = TP/(TP+FP) where TP stands for true positives, FP for false positives, TN for true negatives and FN for false negatives.

We first compared the performance of the classifier genes that have significantly high minimum distances (p-value ≤0.05) with all classifiers (Table 3). The former group performs only slightly better (The simulations performed on different initial sample sizes confirmed this conclusion, data not shown) probably because the starting set (1940 genes) in breast cancer is pre-selected by discriminant analysis. The results suggest that the markers inferred using sample numbers comparable to RCC would still provide a very high degree of separation, even when more samples are used.

More direct testing was carried out by selecting different initial number of samples to compare the performance of the method with 18 samples, to other samples sizes, ranging from 8 (4 normal, 4 tumor) to 24 samples (12 normal, 12 tumor). In each case, we recorded the number of classifiers, number of significant classifiers (p-value ≤0.05) and the performance of the significant classifiers on the test samples (samples not selected initially to identify classifier genes). The number of significant genes were projected (Supplementary Figure 2(a)) based on the results of breast cancer simulations (inset). Overall, the number of classifiers and significant classifiers decreases as the number of samples increases. The performance of significant breast cancer markers (p ≤ 0.05) is shown in (b) and (c). Percentage of correctly classified samples increases with the sample size, having a plateau near 18 samples (b). The values for percentage of misclassified samples and unclassified samples obtained for 18 samples are very close to >20 samples, but much better with respect to lower sample sizes. Sensitivity increases with the sample size, but specificity and PPV of the significant classifiers are independent of the number of samples. Hence, in all cases, the significant classifiers perform well on the test samples in terms of sensitivity and PPV irrespective of how many samples were used to select those classifiers. Overall, the performance of classifiers obtained with 18 samples is better than obtained with smaller number of samples (12, 14 or 16) and almost the same with larger number of samples (≥20 samples).

### 3.4. RCC substructure

We organized the markers into genetic networks using hierarchical clustering (HCL) and principal component analysis (PCA). HCL gives the distances between nodes (samples or genes) and reveals the substructure within nodes; PCA identifies the primary axes upon which the samples vary and how the samples are distributed along these axes based on the similarity between their expression profiles.

#### 3.4.1. HCL

**Figure 3.**
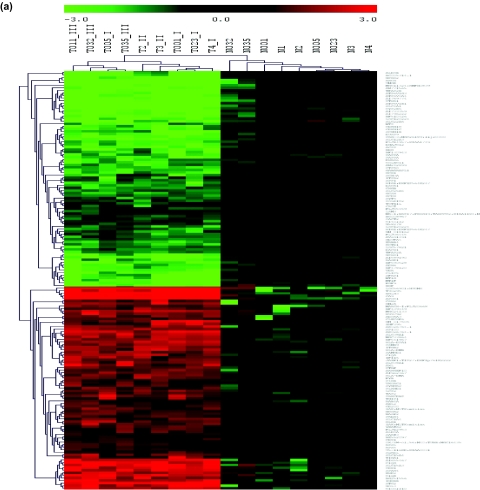

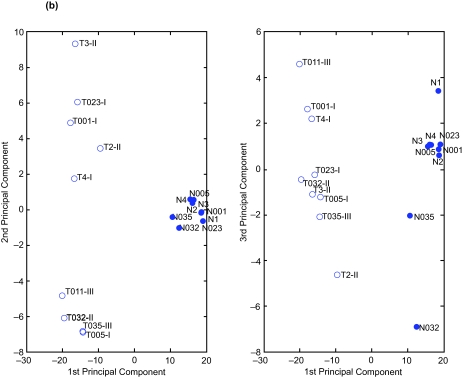
**(a)** Clustering of samples and 158 significant markers using hierarchical clustering. The expression values are normalized for each gene by dividing every expression value to the mean of normal values for that gene and then transforming those values to logarithmic values (log2) to emphasize up or down-regulation with respect to normal expression values. Black represents the mean of normal values, green represents down-regulation and red represents up-regulation with respect to the mean. Clustering of genes reveals two big clusters: down-regulated genes in RCC (upper half) and up-regulated in RCC (bottom half) with respect to the normal samples, together with subclusters within these groups. The markers cluster the samples perfectly well into two major groups (Fig 3a, upper dendrogram). It is clear that tumor samples are separated into two major sub-clusters according to the grade. The only exception is T005 sample (grade I) which clusters with high grade samples. Within the normal samples, N032 and N035 have expression profiles most similar to tumor samples. **(b)** The projection of the samples onto the first three principal components (PC). The eigenvectors of the first three eigenvalues accounted for 86.9% of the variation (81.5, 5.4 and 2.2, respectively) in the data. Tumor samples are represented by open circles; normal samples are shown by filled circles. First principle component separates normal samples from tumor samples while second principle component separates tumor samples with low grade (T3, T023, T001, T2 and T4) from high grade (T011, T032, T035) again with the exception of T005 sample. Third principal component separates normal samples N032 and N035 from the rest of the normal samples as was observed with HCL.

Markers and samples were clustered by average-linkage hierarchical clustering (Figure 3(a)). Each marker is represented as a vector of normalized expression values across all samples and Euclidean distance between vectors was computed. An analogous procedure was used for samples. The distance between two clusters is defined as the distance between pairs of nodes, averaged over all pairs, each pair consisting of one node from each group. At each stage of clustering, the two clusters for which the distance is minimum, are merged. In the resulting dendrograms for samples (top) and genes (left), the height of vertical/horizontal lines are proportional to the degree of similarity between samples/genes.

The expression values are normalized for each gene by dividing every expression value to the mean of normal values for that gene and then transforming those values to logarithmic values (log_2_ transformation) to emphasize up or down-regulation with respect to normal expression values. The expression values are color coded such that red denotes up-regulation with respect to the mean of normal values, green denotes down-regulation and black denotes the mean of normal values. Two big clusters of genes are revealed: down-regulated genes in RCC (upper half) and up-regulated in RCC (bottom half) with respect to the normal samples, together with subclusters within these groups. We didn’t examine these subclusters, but instead analyzed the expression profiles of groups of genes that are in the same pathway in Section 4.1. The markers cluster the samples well into two major groups (Fig. 3a, upper dendrogram). It is clear that tumor samples are separated into two major sub-clusters according to the grade. The only exception is T005 sample (grade I) which clusters with high grade samples for which we cannot provide a satisfactory biological explanation. Within the normal samples, N032 and N035 have expression profiles most similar to tumor samples. Clustering the data with PCA supports these observations as we now explain.

#### 3.4.2. PCA

PCA was applied to the 18 row × 158 column matrix of expression values, normalized as explained above. The eigenvectors of the first three eigenvalues accounted for 86.9% of the variation (81.5, 5.4 and 2.2, respectively) in the data. Figure 3 (b) shows the projection of the samples onto these first three principal components (PCs). The first PC separates normal samples from tumor samples while second PC separates tumor samples with low grade (T3, T023, T001, T2 and T4) from those of high grade (T011, T032, T035) with the exception of T005 sample, as observed with HCL. The third principal component separates normal samples N032 and N035 from the other normal samples. Two of the tumor samples, T3 and T2, are separated from other tumors along the second and third axes, respectively. Both samples are grade II tumors. As expected, PCA suggests that there is more variation within tumor samples than within normal samples, which points to distinct tumor subgroups, reflecting the heterogeneity in cancer phenotype and may have implications for disease progression and response to different therapies.

The separation of N032 and N035 from other normal samples was noted previously (Lenburg et al. 2003) by clustering samples using all 20,192 genes. They observed that N035 clustered with tumor samples; however, we find that it clusters with normal samples as it should. The difference in results reflects our use of markers for clustering. The N032 and N035 profiles are more similar, than other normals, to tumor sample profiles (Figure 3(a)), but still they are more similar to normals than to tumor. Both samples are from RCC patients with grade III tumors, hence there is a possibility that these samples, which were classified as normal by standard histology, are actually a mixture of normal and cancerous tissue.

## 4. Discussion

### 4.1. Pathway associations of markers

Biological roles of 158 markers (Supplementary Table 1) were determined using DAVID (Dennis et al. 2003) and MatchMiner (Bussey et al. 2003) databases. 115 (73%) of the markers (Table 4) are annotated in a KEGG pathway or a GO process at level 5 or higher. Genes that have been identified as having noteworthy differential gene expression in four or more RCC studies (Young et al. 2001; Boer et al. 2001; Takahashi et al. 2001; Gieseg et al. 2002; Lenburg et al. 2003) are indicated with **. The 64 genes implicated in RCC in this study that were not reported by Lenburg et al. are indicated in italic.

Disease association was obtained from OMIM and the Genetic association databases. As summarized in Table 4, 43 are cancer related. The relation between the cancer associated markers previously identified—as well as those identified by this study—and critical physiological changes associated with tumor development (Hanahan and Weinberg, 2000) is also shown in Table 4 (third column, also see Figure 5). In particular column 3 includes (1) genes that were previously implicated in cancer e.g. tumor suppressors and oncogenes and (2) genes that were not previously found to be associated and whose role in transformative processes has not been established. As an example of the latter, we annotated proteolysis genes and genes involved in cell-adhesion and/or regulation of actin cytoskeleton as potentially involved in “tissue invasion and metastasis.”

**Figure 4. (a-f).**
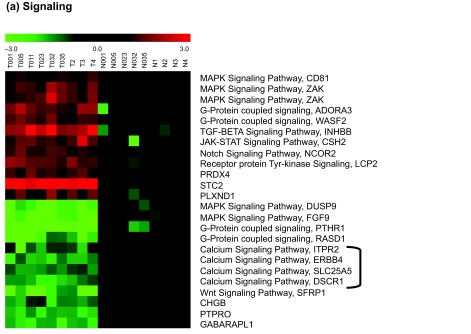

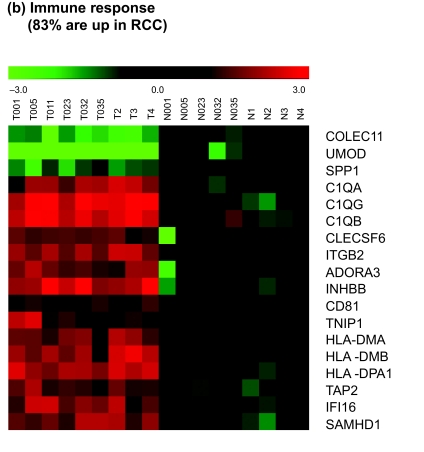

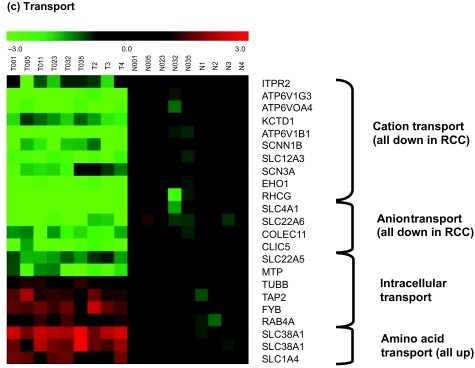

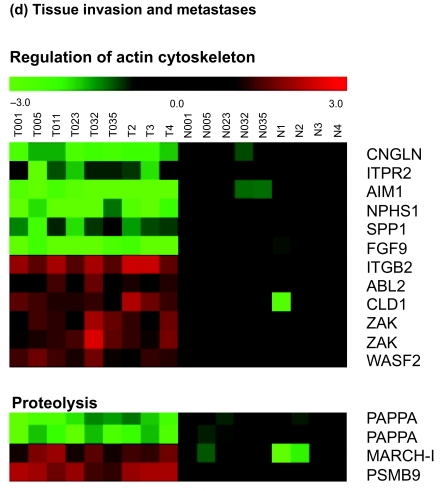

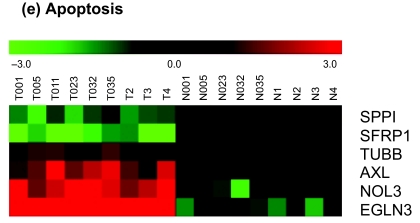

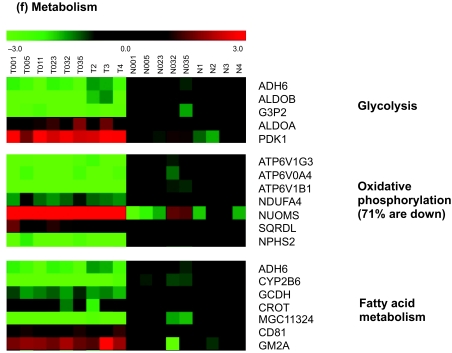
Heatmaps of some of the important pathways that 158 markers are involved in. The expression values for each gene are transformed (Section 3.4.1) and color coded as in Figure 3. Black represents the mean of normal values, green represents down-regulation and red represents up-regulation with respect to the mean.

As summarized in Table 4, the markers include several signaling proteins, some of them previously implicated in cancer transformation (Notch signaling, Wnt signaling, TGF-β signaling, NF-κB cascade and MAPK signaling cascades). The heatmap of signaling pathway genes is shown in Figure 4(a). *Interestingly, all markers involved in calcium signaling pathways are down-regulated in RCC. Another interesting group of genes are immune system related genes, 83% of which are upregulated* (Figure 4(b)). Considering that RCC is resistant to chemo, radio and hormonal therapy while immunotherapy (cytokines IL-2 and interferon-alpha) appears to be effective for RCC treatment, these immune response related biomarkers could be potentially important for therapeutics. Our results also indicate that all cation and anion transport genes identified by our analysis—which encode mainly ion channels—are down-regulated (Figure 4(c)). Conversely, genes involved in intracellular transport and amino acid transport are mostly up-regulated. Since the kidney is at the junction of circulatory and urological processes both of which involve transport of different body fluids as well as ions; down-regulation of ion channels may be the result of a nonfunctioning kidney in RCC. Increase in intracellular transport point to increased communication of cancerous cells with ECM, with normal cells as well as with other cancer cells. Up-regulated amino acid transport shows that cancer cells need more building blocks to make proteins for different biological processes as they grow.

Other groups of genes potentially important for tumor progression include those for proteolysis, and cell-adhesion and/or regulation of actin cytoskeleton (Figure 4(d)). These genes are likely to be involved in tissue invasion and metastasis.

The apoptotic genes (Figure 4(e)) SPP1 and SFRP1 are candidate tumor suppressors for various cancers; AXL gene is a proto-oncogene, and NOL3 (ARC, apoptosis repressor with CARD domain) is induced in human breast cancer and confers chemo- and radiation-resistance (Mercier et al. 2005). These genes may all contribute to evasion of apoptosis during tumor development.

Finally, a number of markers are involved in metabolism; some are shown in Figure 4(f). It is clear that the processes shown are all linked to energy generation in the cell and that most of these genes are suppressed in RCC. Studies by Warburg (Warburg, 1956) indicate that the vast majority of human and animal tumors display a high rate of glycolysis under aerobic conditions. Human solid tumors endure profound hypoxia; hence adaptation to hypoxic conditions is a crucial step in tumor progression. The anaerobic use of glucose as an energy source through glycolysis is therefore a feature common to solid tumors, in turn leading to a lesser dependence on the mitochondria for oxidative phosphorylation. This loss of cell dependence on oxidative metabolism is an important factor in the development of tumors. In accordance with that, it was shown that expression levels of OXPHOS genes were down-regulated in RCC (Simonnet et al. 2002; Meierhofer et al. 2004). Hence, overall suppression of these genes can be due to loss of tumor dependence on normal energy generating pathways in hypoxic conditions.

### 4.2. Disease associations

Eleven of the 158 biomarkers are especially definitive, both because of their biology, and because they have been identified as RCC related in four or more studies (Young et al. 2001; Boer et al. 2001; Takahashi et al 2001; Gieseg et al. 2002; Lenburg et al. 2003). These genes are GABARAPL1, EGLN3, MT1G, SFRP1, INHBB, ATP6V1B1, APOC1, ADH6, C1QB, ALDOB and CNGLN. SFRP1 and EGLN3 are involved in apoptosis, which is a critical process as evasion of apoptosis is one of the key steps in tumorigenesis. CNGLN is involved in regulation of actin cytoskeleton, which may have a role in tissue invasion and metastasis. Four other genes have a role in metabolism, specifically glycolysis (ADH6, ALDOB), lipoprotein metabolism (APOC1), and oxidative phosphorylation (ATP6V1B1). INHBB and C1QB are immune response related genes. INHBB is a growth factor, hence its up-regulation may cause uncontrolled activation of downstream targets.

In addition to these eleven genes, 83 others were identified by Lenburg et al. as differentially expressed in RCC. They include carbonic anyhdrase IX, which is the RCC associated antigen G250 and is induced in many cancer types, hypoxia induced gene ADORA3, potentially oncogenic AXL gene which causes transformation when overexpressed in NIH 3T3 cells, and vitamin D receptor (VDR, up-regulated), which was found to be over-expressed in pancreatic cell lines (Albrechtsson et al. 2003) and is down-regulated by resveratrol compound (Shi et al. 2004) in RCC cell lines, which acts as a chemopreventive agent for RCC and other types of cancers.

Another 64 genes were not identified in our previous study. Of these genes 25 were found to be significantly up-/down-regulated (in the same direction with this study) by one or more other RCC studies as summarized in Table 5. The overall summary of the 7 RCC studies and the overlap between their gene sets and our genes are given in Supplementary Table 2.

TUBB, PRDX4 and ZNF395 genes are potentially important genes since they have been identified by four or more RCC studies including us. Some of the genes were already shown to be important in RCC by additional studies other than listed in Table 5. These genes include SLC25A5 (ANT2, down), which catalyzes the exchange of ATP for ADP across the mitochondrial membrane, thus playing an important role in oxidative phosphorylation. Renal carcinomas were found to have reduced levels of ANT2 and other oxidative phosphorylation genes (Heddi et al. 1996) in line with the argument that the loss of cell dependence on oxidative metabolism is an important factor in the development of tumors under hypoxic conditions (Chevrollier et al. 2005). ALDOA enzyme (up), originally found to be up-regulated in lung cancer (Ojika et al. 1991), was determined to be an indicator of poor prognosis in RCC patients in combination with gamma-enolase (Takashi et al. 1993).

We further classified 64 markers into three groups (Table 6) based on their disease associations: (I) previously reported RCC related genes (Table 5), (II) genes related to cancers other than RCC, and (III) genes related to diseases other than cancer.

(II) These genes include NCOR2 (SMRT, up), which forms a large co-repressor complex that contains SIN3A/B and histone deacetylases HDAC1 and HDAC2. This complex associates with the thyroid (TR) and the retinoid acid receptors (RAR) in the absence of ligand, and may stabilize their interactions with TFIIB. Recently, it has been shown that elevated SMRT levels result in suppression of target genes for the vitamin D receptor (VDR) in prostate cancer cells and in apparent hormonal insensitivity (Khanim et al. 2004). ABL2 (up) tyrosine kinase is related to proto-oncogene ABL, and is implicated in hematologic neoplasms (Yagasaki et al. 2001) and gastric adenocarcinoma (Wu et al. 2003).

CD81 antigen (up) is reported to influence adhesion, morphology, activation, proliferation, and differentiation of B, T, and other cells. Antibodies against CD81 induce homotypic aggregation of cells and can inhibit their growth. The loss of CD81 was found to be associated with differentiation and metastasis of HCC (Inoue et al. 2001). Betta-tubulin, TUBB, gene is implicated in many cancers including ovarian and lung cancer. Non-small cell lung cancers have a high incidence of somatic mutations of the beta-tubulin (class I) gene, which may cause paclitaxel resistance (de Castro et al. 2003). Moreover, recently, class III beta-tubulin overexpression was found to be a prominent mechanism of paclitaxel resistance in ovarian cancer patients (Mozzetti et al. 2005).

SART3 (squamous cell carcinoma antigen recognized by T cells 3), is an RNA-binding nuclear protein that is a tumor-rejection antigen. This antigen possesses tumor epitopes capable of inducing HLA-A24-restricted and tumor-specific cytotoxic T lymphocytes in colorectal cancer patients and may be useful for specific immunotherapy (Sasatomi et al. 2002). TFAP2 is known to suppress a number of genes including MCAM/MUC18, C/EBP alpha and MYC. The loss of this gene was shown to be to be associated with malignant transformation and tumor progression in malignant melanoma (Karjalainen et al. 1998; Jean et al. 1998). This gene may be a potential tumor suppressor protein for RCC as well.

(III) Markers related to diseases other than cancer include WASF2, LCP2 and FYB (Wiscott-Aldrich syndrome, all up) and TAP2 (up, polymorphisms in this gene are implicated in type 1 diabetes (Lotfi et al. 1994; Penfornis et al. 2002). The relationship between Wiscott-Aldrich syndrome and diabetes should be elaborated in the future.

Other genes which we’ve identified as RCC related, but which have not previously been associated with any disease include ITPR2, which has roles in the calcium and phosphatidylinositol signaling pathways; glutaryl-Coenzyme A dehydrogenase (GCDH), which takes part in fatty acid metabolism,; 40S ribosomal protein S5 (RPS5), HIG1 (likely ortholog of hypoxia induced gene 1) and P4HB (proline 4-hydroxylase).

### 4.3. Genes related to critical processes underlying kidney cell transformation

The development of RCC, as with other cancers, is accompanied by alterations in cell physiology, which collectively dictate malignant growth (Hanahan and Weinberg, 2000). Briefly these changes include environment independent growth; insensitivity to antigrowth factors (loss of tumor suppressor genes); evasion of apoptosis (producing survival factors); limitless replicative potential (turning on telomerase); sustained angiogenesis (producing VEGF inducer) and tissue invasion and metastasis (inactivation of E-cadherin). The order in which these capabilities are acquired is likely to be variable across different cancer types and subtypes. In this section we discuss RCC markers in the context of these alterations.

**Figure 5.**
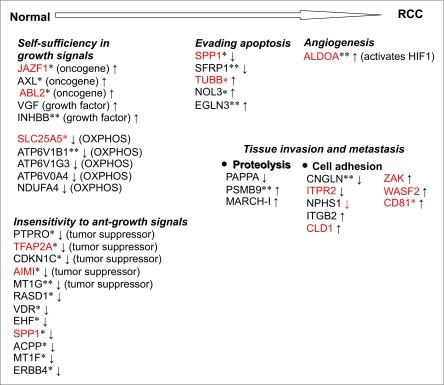
Genes related to critical processes underlying kidney cell transformation. Marker genes were replaced into six Weinberg categories which are essential for tumor development. Genes previously found by at least four other RCC studies are indicated with **, genes implicated in other cancers with *, and markers not identified previously by Lenburg et al. are given in red. Since the exact order of these steps is not known, the processes are given in here with no particular order.

The main associations for RCC are summarized in (Figure 5 and Table 4). We include (1) genes that were previously implicated in cancer e.g. tumor suppressors and oncogenes and (2) genes that were not previously found to be associated with cancer but which have a function critical to tumor development. We did not include the genes whose expression is not correlated with the associated category e.g. NCOR2 gene (Table 4) suppresses tumor growth in prostate cancer cells but is up-regulated in RCC, and hence it is not included. Our results suggest the following for RCC (1) Self-sufficiency in growth signals is achieved via activation of oncogenes JAZF1, AXL, ABL2; and growth factors INHBB and VGF. Further, loss of OXPHOS genes SLC25A5, ATP6V1B1, B3, V0A4, and NDUFA4 may contribute to the self-sufficiency of the cancer cells with the ability to be less dependent on OXPHOS (2) The loss of tumor suppressor genes PTPRO, TFAP2A, CDKN1C, AIM1 and MT1G as well as other genes that were shown to suppress tumor growth in cancer cell lines but not yet identified as tumor suppressor candidates (RASD1, VDR, EHF, SPP1, ACPP, MT1F and ERBB4) contributes to insensitivity to antigrowth signals; (3) Evasion of apoptosis is mediated through loss of SPP1 and SFRP1, and activation of TUBB, NOL3 and EGLN3. (4) Two groups of genes are likely to be involved in tissue invasion and metastasis: proteolysis genes (PAPPA, PSMB9 and MARCH-1) and genes involved in cell-adhesion and/or regulation of actin cytoskeleton (CNGLN, ITPR2, NPHS1, ITGB2, CLD1, ZAK, WASF2, CD81) and (5) Angiogenesis may be mediated through ALDOA enzyme which is shown to be activated by HIF1 under hypoxic conditions and by increased glycolytic activity (Warburg effect), and which in a feedback loop activates HIF1 (Lu et al. 2002) which then activates several angiogenic factors including VEGF.

The identification of these genes opens up many paths for investigation that would not otherwise have been apparent. For example down regulation is often the result of epigenetic modification of upstream regions; especially methylation. The identification of CpG islands in or around binding sites and their analysis by RT-PCR or MALDI-TOF would be an obvious route to take, and if significant methylation difference are found, it would suggest a simple and sensitive assay for potentially significant markers.

## Supplementary

Supplemental Figure 1. Number of occurrences of sorted genes in top 20 rankings. The highest ranked gene, ie gene #1, has 17 occurrences across 18 top 20 lists.

Supplemental Figure 2. Simulations on breast cancer dataset (Ma et al. 2003) with different number of samples. **(a)** The number of significant genes projected for different number of samples based on the results of breast cancer simulations (inset). **(b)** and **(c)** Performance of significant breast cancer markers (p ≤ 0.05) on the test samples.

**Supplementary Table 1.** 158 significant (p-value ≤0.01) markers.

**GenBank Accession**	**Symbol**	**p-value**
NM_003221	TFAP2B	0
NM_005235	ERBB4	0
NM_017753	PRG-3	0
NM_003714	STC2	1x10 ^−5^
AI655467		2x10 ^−5^
BF478120		2x10 ^−5^
NM_001692	ATP6V1B1	2x10 ^−5^
NM_002489	NDUFA4	2x10 ^−5^
NM_012232	PTRF	2x10 ^−5^
NM_021179	LOC57821	2x10 ^−5^
NM_031412	GABARAPL1	2x10 ^−5^
AI733359		3x10 ^−5^
NM_005950	MT1G	3x10 ^−5^
NM_006990	WASF2	3x10 ^−5^
NM_172369	C1QG	3x10 ^−5^
BF541967		5x10 ^−5^
NM_002010	FGF9	5x10 ^−5^
NM_033554	HLA-DPA1	5x10 ^−5^
AI589190		6x10 ^−5^
BC005314.1		6x10 ^−5^
NM_004646	NPHS1	6x10 ^−5^
NM_133262	ATP6V1G3	6x10 ^−5^
NM_174896	MGC24133	6x10 ^−5^
NM_002848	PTPRO	7x10 ^−5^
NM_003113	SP100	7x10 ^−5^
NM_014625	NPHS2	7x10 ^−5^
NM_000339	SLC12A3	8x10 ^−5^
NM_000491	C1QB	9x10 ^−5^
NM_001009	RPS5	9x10 ^−5^
NM_000767	CYP2B6	0.00011
NM_003012	SFRP1	0.00011
NM_004894	C14orf2	0.00011
NM_016929	CLIC5	0.00011
NM_022073	EGLN3	0.00011
NM_033201	BC008967	0.00011
NM_004356	CD81	0.00013
NM_138799	OACT2	0.00013
NM_000211	ITGB2	0.00014
AK026764.1		0.00015
NM_152522	MGC33864	0.0003
AV691491		0.00033
NM_004392	DACH1	0.00033
NM_005565	LCP2	0.00033
NM_014463	LSM3	0.00033
NM_015474	SAMHD1	0.00036
BG251556		0.00037
NM_018162	HELAD1	0.0004
NM_000376	VDR	0.00043
NM_001819	CHGB	0.00047
NM_020142	NUOMS	0.00047
NM_004578	RAB4A	0.00055
AI962367		0.00058
NM_021800	DNAJC12	0.0006
NM_021199	SQRDL	0.00061
NM_153233	FLJ36445	0.00073
NM_017606	NM_017606	0.0008
NM_015488	MR-1	0.00084
BF439449		0.00093
NM_000342	SLC4A1	0.00093
NM_006120	HLA-DMA	0.00098
NM_000918	P4HB	0.00108
NM_001099	ACPP	0.00113
NM_021151	CROT	0.00113
BG434272		0.00121
NM_001216	CA9	0.00122
NM_198991	KCTD1	0.00125
NM_006312	NCOR2	0.00126
NM_016582	SLC15A3	0.00142
NM_020632	ATP6V0A4	0.00142
NM_003220	TFAP2A	0.00166
NM_005158	ABL2	0.00169
NM_014601	EHD2	0.00169
NM_003116	SPAG4	0.00185
AW771565	AIM1	0.00187
NM_003946	NOL3	0.00192
NM_000076	CDKN1C	0.00195
NM_006058	TNIP1	0.00197
NM_000336	SCNN1B	0.00198
NM_000035	ALDOB	0.00202
NM_015103	PLXND1	0.00206
BF130943		0.00217
BE552097		0.00222
NM_000672	ADH6	0.00248
BE739519		0.00259
NM_198446	FLJ45459	0.00259
NM_021958	HLX1	0.0026
NM_001395	DUSP9	0.00262
NM_018023	YEATS2	0.00267
NM_001004196	CD200	0.00269
NM_006520	TCTE1L	0.00275
NM_001152	SLC25A5	0.00276
NM_002193	INHBB	0.00277
NM_006922	SCN3A	0.00277
NM_000159	GCDH	0.0029
NM_002800	PSMB9	0.00314
NM_004051	BDH	0.00314
NM_145040	PRKCDBP	0.0032
N58278		0.00325
NM_024006	VKORC1	0.00335
NM_004710	SYNGR2	0.00339
AI796222		0.00342
NM_000161	GCH1	0.00348
NM_000544	TAP2	0.00357
NM_014706	SART3	0.00357
NM_014056	HIG1	0.00362
NM_001645	APOC1	0.00364
NM_012153	EHF	0.00364
NM_175061	JAZF1	0.00368
NM_015991	C1QA	0.00379
NM_145648	SLC15A4	0.00384
NM_178014	TUBB	0.00391
NM_000405	GM2A	0.00392
AW242899		0.00407
NM_000582	SPP1	0.00408
NM_002610	PDK1	0.00412
NM_007021	C10orf10	0.00413
NM_016084	RASD1	0.00423
NM_016184	CLECSF6	0.00433
NM_017923	MARCH-I	0.00438
NM_015584	POLDIP2	0.00456
NM_006406	PRDX4	0.00468
NM_020991	CSH2	0.0047
NM_000677	ADORA3	0.00478
NM_002223	ITPR2	0.00483
BF590528		0.00485
NM_005949	MT1F	0.00489
NM_003038	SLC1A4	0.0049
NM_001465	FYB	0.00503
NM_004790	SLC22A6	0.00514
NM_024027	COLEC11	0.00514
AI769774		0.00525
NM_016653	ZAK	0.00525
NM_014629	ARHGEF10	0.00527
NM_000253	MTP	0.00571
NM_003361	UMOD	0.00576
BF510426		0.00583
NM_005531	IFI16	0.006
AI282982	LOC120224	0.00629
NM_004247	U5-116KD	0.00634
NM_032118	FLJ12953	0.00641
NM_004414	DSCR1	0.00655
NM_032866	CNGLN	0.00665
NM_002118	HLA-DMB	0.00719
NM_004483	GCSH	0.00742
NM_000316	PTHR1	0.00743
T90295		0.0076
NM_030674	SLC38A1	0.00765
NM_001699	AXL	0.00773
AW242836	LOC120224	0.0078
NM_205848	SYT6	0.00895
NM_000034	ALDOA	0.00896
NM_032717	MGC11324	0.00942
NM_020139	DHRS6	0.00945
AA148534	PAPPA	0.00951
NM_016321	RHCG	0.00956
H99792		0.00983
NM_053000	TIGA1	0.00983
NM_018660	ZNF395	0.00994

**Supplementary Table 2.** Comparison with other RCC studies.

	**Method**	**number of samples/microarray platform**	**Final number of genes**	**Overlap with 158 marker genes**	Overlap with 64 * marker-genes
Young et al. 2001	More than two-fold changed in two or more tumor samples	7 tumor (4 cc-RCC), 7 normal/cDNA 7,075 genes	189	8	1
Takahashi et al. 2001	Three-fold or more changed in 75% or more of the tumor samples	29 cc-RCC and 29 normal/cDNA 21,632 genes	109	7	-
Gieseg et al. 2002	Changed genes in cc-RCC with Wilcoxon test p-value ≤0.001 and fold change ≥1.1	13 RCC (9cc-RCC), 9 normal/ Affymetrix 5600 genes	355 genes, 85 reported	4	1
Boer et al. 2001	adapted sign test by counting for each gene the number of times that its measured intensity in the set of repeated pair-wise comparisons is higher in T and N	37 cc-RCC, 37 normal	1738 cDNAs	45	11
Higgins et al. 2003	No reported gene set; selected genes with avg fold change >3 and t-test p-value >0.03.	41 RCC (23 cc-RCC), 3 normal/cDNA 22,648 genes	182 genes	8	1
Jones et al. 2005	90% lower confidence boung of the fold change was >2 and t-test p-value <0.001	8 clear cell stage I, 23 normal, Affymetrix 22,283 genes	1359 up-regulated, 493 down-regulated	37 up, 19 down-regulated	15 up-regulated
Sultmann et al. 2005	t-test with estimated false discovery rate <0.23	25 ccRCC, 25 normal/RCC-specific cDNA microarrays with 4207 genes	620 up-regulated; 561 down-regulated genes	13 up, 11 down-regulated	5 up, 2 down-regulated

* genes not identified by Lenburg et al.

## Figures and Tables

**Table 1.** Top ranked pathways with percentage of significant classifier genes.

**Pathway**	**Number of classifier genes**	**Number of classifier genes/number of genes in the pathway (%)**
Glycolysis	5	12.5
Antigen processing	4	11.76
Oxidative phosphorylation	5	3.36
Calcium signaling pathway	4	3.13
G-Protein coupled signaling	4	2.99
MAPK signaling pathway	4	2.06
Immune response	14	1.71
Fatty acid metabolism	4	1.53
Cation transport	6	1.19
Apoptosis	6	0.98
Intracellular transport	6	0.95
Regulation of transcription	12	0.58
Cell adhesion	4	0.56

**Table 2.** Top 20 ranked genes by SVM and their significance as classifier by exhaustive search.

**SVM rank**	**Gene**	**p-value (min dist)**	**Min dist rank**	**Related disease**
1	ALDOB**	6.00E-05	23	RCC
2	NDUFA4	2.00E-05	8	
3	TFAP2B*	0	1	tumor suppressor candidate, melanoma
4	TCTE1L	0.00275	93	
5	LOC57821	2.00E-05	11	
6	GABARAPL3*†	0.02892	239	
7	SLC38A1*	7.00E-05	27	
8	POLDIP2	0.00456	124	
9	DACH1	0.00033	43	
10	GABARAPL1**	2.00E-05	9	RCC
11	HLA-DPA1*	5.00E-05	16	Melanoma
12	ERBB4*	0	2	Breast, ovarian cancer
13	HIG1	0.00362	108	hypoxia induced
14	EHD2*	0.00169	74	
15	CD81	0.00013	38	Hepatoma
16	PRG-3*	0	3	
17	NPHS1*	6.00E-05	19	Non cancerous kidney diseases
18	C1QA*	0.00379	112	
19	ZNF697†	0.01543	191	
20	PIGR*†	0.38576	432	

^†^: minimum distance p-value >0.01

** identified by four or more RCC studies (Young et al. 2001; Boer et al. 2001; Takahashi et al. 2001; Gieseg et al. 2002; Lenburg et al. 2003)

* identified by Lenburg et al.

**Table 3.** Performance of classifiers on the test samples in breast cancer dataset with 18 initial samples.

	**Classifiers with p-value ≤0.05**	**All classifiers**
% correctly classified samples	88%	82%
% misclassified samples	11%	18%
Sensitivity	0.87	0.8
Specificity	0.92	0.85
PPV	0.96	0.91

**Table 4.** Biological roles (from KEGG and GO databases) and disease associations of 115 annotated gene markers.

**Down-regulated genes**			
**Gene**	**Related disease**	**Weinberg category**	**Pathway**
DUSP9			MAPK Signaling/JNK cascade
FGF9	Prostate, ovarian cancer	Self-sufficiency in growth signals	MAPK Signaling/Regulation of actin cytoskeleton/growth factor
*ITPR2*		Tissue invasion and metastasis	Calcium Signaling Pathway/Gap junction
ERBB4	Breast, ovarian cancer	Insensitivity to anti-growth signals	Calcium Signaling Pathway
*SLC25A5*	RCC	Self-sufficiency (Loss of cancer cell dependence on OXPHOS)	Calcium Signaling Pathway/Intracellular transport
DSCR1			Calcium Signaling Pathway
PTHR1	Chronic kidney failure		G-Protein coupled signaling
RASD1	Suppresses cell growth in human breast cancer and lung cancer cell lines	Insensitivity to anti-growth signals	G-Protein coupled signaling
SFRP1**	RCC, bladder cancer, cervical cancer	Evasion of apoptosis	Wnt Signaling Pathway/Apoptosis
*CHGB*	Neuroendocrine tumors		Signaling/hormone
PTPRO	Lung cancer	Insensitivity to anti-growth signals	Signaling/tumor suppressor candidate
GABARAPL1**	RCC		Signaling
ATP6V1G3		Self-sufficiency (Loss of cancer cell dependence on OXPHOS)	Oxidative phosphorylation
ATP6V0A4		Self-sufficiency (Loss of cancer cell dependence on OXPHOS)	Oxidative phosphorylation
ATP6V1B1**	RCC/renal tubular acidosis	Self-sufficiency (Loss of cancer cell dependence on OXPHOS)	Oxidative phosphorylation
KCTD1			Cation transport
SCNN1B			Cation transport
SLC12A3			Cation transport
SCN3A			Cation transport
EHO1			Cation transport
RHCG			Cation transport
SLC4A1			Anion transport
SLC12A3			Anion transport
SLC22A6			Anion transport
COLEC11			Anion transport/Immune response
CLIC5			Anion transport
MTP			Intracellular transport
*DACH1*			Transcription regulation
TFAP2B	Char syndrome		Transcription regulation
*TFAP2A*	Melanoma	Insensitivity to anti-growth signals	Transcription regulation/tumor suppressor candidate
VDR	RCC	Insensitivity to anti-growth signals	Transcription regulation
EHF	Prostate, breast, and lung carcinomas	Insensitivity to anti-growth signals	Transcriptional repressor
*LSM3*			RNA splicing
RCQ5			DNA repair
*HELAD1*	Up in colorectal cancer	Limitless replicative potential	DNA replication
PAPPA		Tissue invasion and metastasis	Proteolysis
DNAJC12			Protein folding
CNGLN**	RCC	Tissue invasion and metastasis	Regulation of actin cytoskeleton
AIM1	Melanoma	Insensitivity to anti-growth signals+Tissue invasion and metastases	Cell adhesion/ tumor suppressor candidate
NPHS1	Kidney diseases	Tissue invasion and metastasis	Cell adhesion
*SPP1*	down-regulated in RCC and intrahepatic cholangiocarcinoma; up-regulated in breast, prostate, colon (and others) carcinomas	Insensitivity to anti-growth signals	Cell adhesion/Apoptosis/Immune response
CDKN1C	Breast, pancreatic, thyroid cancer	Insensitivity to anti-growth signals	Cell cycle/tumor suppressor gene
UMOD			Immune response
ADH6**	RCC		Glycolysis/Fatty acid metabolism
ALDOB**	RCC, hepatocellular carcinoma		Glycolysis
G3P2			Glycolysis
CYP2B6	Breast cancer		Fatty acid metabolism
*GCDH*			Fatty acid metabolism
*CROT*			Fatty acid metabolism
MGC11324			Membrane lipid metabolism
BDH			Synthesis and degradation of ketone bodies
*NDUFA4*		Self-sufficiency (Loss of cancer cell dependence on OXPHOS)	Oxidative phosphorylation
NPHS2	Kidney diseases		Energy metabolism
GCSH			Amino Acid metabolism
GCH1			Amino Acid metabolism
ACPP	Reduces cell growth in prostate cancer upon induction by Vitamin D receptor agonists	Insensitivity to anti-growth signals	Metabolism
DHRS6			Metabolism
*HIG1*			Hypoxia induced gene
MT1G**	RCC, papillary thyroid carcinoma, prostate cancer	Insensitivity to anti-growth signals	Metallothionein gene/tumor suppressor candidate
MT1F	Breast, liver cancer. Suppresses growth of liver cell line HepG2	Insensitivity to anti-growth signals	Metallothionein gene

**Up-regulated genes**			
**Gene**	**Related disease**	**Weinberg category**	**Pathway**
*CD81*	Hepatoma	Tissue invasion and metastasis	MAPK Signaling/Immune response/Membrane lipid metabolism/Cell adhesion
*ZAK*		Tissue invasion and metastasis	MAPK Signaling/Tight junction/Cell cycle
ADORA3	ADORA3 agonists inhibit growth in leukemia and breast cancer cell lines	Insensitivity to anti-growth signals	G-Protein coupled signaling/Immune response/Hypoxia induced gene
*WASF2*	Wiscott-Aldrich syndrome	Tissue invasion and metastasis	G-Protein coupled signaling/Adherens junction
*NCOR2*	Suppresses antiproliferative targets of VDR in prostate cancer	Insensitivity to anti-growth signals	Notch Signaling Pathway/transcriptional repressor
CSH2			JAK-STAT Signaling Pathway
*LCP2*	Wiscott-Aldrich syndrome		Receptor protein Tyr-kinase Signaling
INHBB**	RCC	Self-sufficiency in growth signals	TGF-β Signaling Pathway /Immune response/growth factor
STC2	Breast cancer		Signaling/hormone/renal and intestinal calcium and phosphate transport
*PRDX4*			NF-κB cascade
*PLXND1*			Signaling
C1QA			Immune response
C1QG			Immune response
C1QB**	RCC		Immune response
SLC38A1			Amino Acid Transport
*SLC1A4*			Amino Acid Transport
*TUBB*	Ovarian, lung cancer	Evasion of apoptosis	Intracellular transport/Apoptosis
*TAP2*	Type 1 diabetes		Intracellular transport/Antigen processing
*FYB*	Wiscott-Aldrich syndrome		Intracellular transport
*RAB4A*			Intracellular transport
*YEATS2*			Transcription regulation
*PTRF*			Transcription regulation
SP100	Liver diseases		Transcription regulation
*JAZF1*	Endometrial stromal tumors	Self-sufficiency in growth signals	Transcription regulation/oncogene
IFI16	Head and neck squamous cell carcinomas. Up-regulation inhibits tumor growth	Insensitivity to anti-growth signals	Transcription regulation/ Antigen processing
*HLX1*			Transcription regulation
*ZNF395*			Transcription regulation
*RPS5*			Protein biosynthesis
FUT11			Protein biosynthesis
*U5-116KD*			RNA splicing
NOL3	Breast cancer	Evasion of apoptosis	RNA splicing/anti-apoptotic
*SART3*	Colorectal cancer		RNA processing
PSMB9	RCC	Tissue invasion and metastasis	Proteolysis
MARCH-I		Tissue invasion and metastasis	Ubiquitin cycle
ITGB2		Tissue invasion and metastasis	Cell adhesion/Immune response
*ABL2*	Gastric adenocarcinoma	Self-sufficiency in growth signals + Tissue invasion and metastases	Focal adhesion/oncogene
*CLD1*		Tissue invasion and metastasis	Tight junction
AXL	RCC	Self-sufficiency in growth signals + Evasion of apoptosis	Apoptosis/oncogene
EGLN3**	RCC	Evasion of apoptosis	Apoptosis
CLECSF6			Immune response
*TNIP1*			Immune response
*HLA-DMA*			Antigen processing
HLA-DMB			Antigen processing
HLA-DPA1	Melanoma		Antigen processing
*SAMHD1*			Interferon induction
PDK1			Glycolysis/Gluconeogenesis
*ALDOA*	RCC, HIF1 activated gene, also activates HIF1	Increased glycolysis (Warburg effect) + Angiogenesis	Glycolysis/Gluconeogenesis
GM2A			Membrane lipid metabolism
*VKORC1*			Biosynthesis of steroids
NUOMS			Oxidative phosphorylation
CA9	RCC		Nitrogen metabolism
*SQRDL*			Energy metabolism
APOC1**	RCC		Lipoprotein metabolism
*P4HB*	May be HIF1 related		Metabolism
VGF		Self-sufficiency in growth signals	Growth factor

Genes in italic: 64 genes previously not reported by Lenburg et al.

** genes previously reported by 4 or more RCC studies

**Table 5.**
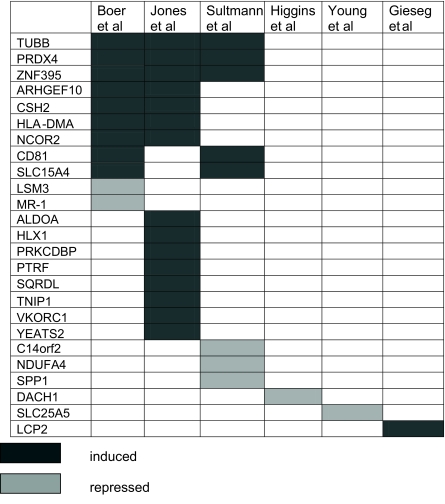
25 genes that were not identified by Lenburg et al but identified by other RCC studies including this study.

**Table 6.** Disease related 64 markers not identified by Lenburg et al.

**Genes related to cancers other than RCC**		
NCOR2	Up	Suppresses target genes for the vitamin D receptor (VDR) in prostate cancer cells resulting in hormonal insensitivity (Khanim et al. 2004)
ABL2	Up	Related to proto-oncogene ABL. Implicated in hematologic neoplasms (Yagasaki et al. 2001) and gastric adenocarcinoma (Wu et al. 2003)
CD81	Up	The loss of CD81 was found to be associated with differentiation and metastasis of HCC (Inoue et al. 2001)
TUBB	Up	Implicated in many cancers including ovarian (Mozzetti et al. 2005) and lung cancer (de Castro et al. 2003)
SART3	Up	Induces HLA-A24-restricted and tumor-specific cytotoxic T lymphocytes in colorectal cancer patients (Sasatomi et al. 2002)
HELAD1	Down	Up-regulated in colorectal carcinomas (Ishiguro et al. 2002)
CHGB	Down	Up-regulated in neuroendocrine tumors (Kimura et al. 2000)
JAZF1	Up	Frequent fusion of the JAZF1 and JJAZ1 genes in endometrial stromal tumors (Huang et al. 2004)
PRKCDBP	Up	epigenetic or mutational inactivation contribute to the pathogenesis of breast and lung cancers (Xu et al. 2001)
TFAP2A	Down	Loss of AP-2 results in metastasis of melanoma cells (Jean et al. 1998)

**Genes related to diseases other than cancer**		
WASF2	Up	Wiscott-Aldrich syndrome
LCP2	Up	Wiscott-Aldrich syndrome
FYB	Up	Wiscott-Aldrich syndrome
TAP2	Up	Type 1 diabetes
